# PREHABILITATION FOR PATIENTS UNDERGOING METABOLIC/BARIATRIC SURGERY: A RETROSPECTIVE COHORT STUDY USING PROPENSITY SCORE MATCHING

**DOI:** 10.2340/jrm.v58.45736

**Published:** 2026-08-10

**Authors:** Yiling YAN, Shengdi LU, Heming WANG, Jiayuan PENG, Lihua HUANG, Yun SHEN, Feng LIU

**Affiliations:** 1 Department of Gastroenterology, Shanghai Tenth People’s Hospital, Tongji University School of Medicine, Shanghai, China; 2 Pennington Biomedical Research Center, Baton Rouge, LA, USA; 3 Department of Gastroenterology, Qingdao Municipal Hospital, Qingdao; 4 Department of General Surgery, Shanghai Sixth People’s Hospital, Shanghai; 5 Department of Rehabilitation Medicine, Shanghai Sixth People’s Hospital, Shanghai; 6 Digestive Endoscopy Center, Shanghai Tenth People’s Hospital, Tongji University School of Medicine, Shanghai, China

**Keywords:** prehabilitation, bariatric surgery, propensity score matching, cost analysis, rehabilitation

## Abstract

**Importance:**

Multimodal prehabilitation improves outcomes in colorectal surgery, but its effectiveness and cost-effectiveness before metabolic and bariatric surgery are unknown.

**Objective:**

To evaluate the effectiveness and cost-effectiveness of a 6-week multimodal prehabilitation programme compared with standard preintervention education in patients undergoing bariatric surgery.

**Design, setting, and participants:**

Propensity score matched cohort observational study at Shanghai Tenth People’s Hospital, China, January 2022 to January 2025. Sixty prehabilitation patients were matched 1:1 to 60 controls from 254 standard care candidates using nearest-neighbour matching on the logit of the propensity score. Follow-up was 12 months.

**Interventions:**

A 6-week programme of supervised exercise, nutritional counselling, and psychological support vs 2 standard preoperative counselling sessions. All patients underwent Roux-en-Y gastric bypass or sleeve gastrectomy.

**Main outcomes and measures:**

Total weight loss at 12 months. Secondary outcomes included body composition, metabolic parameters, functional capacity, patient-reported outcomes, safety, and healthcare costs.

**Results:**

Among 120 matched patients (mean [SD] age, 32.6 [5.2] years; 65.8% male; mean body mass index [BMI], 38.2 [3.2] kg/m^2^), total weight loss at 12 months did not differ between groups (23.7 [5.1] vs 23.4 [2.7] kg; difference, 0.3 kg; 95% CI, −1.3 to 1.8; *p* = 0.75). At 3 months, prehabilitation showed significantly greater weight loss (adjusted β = 2.85 kg; 95% CI, 1.77 to 3.93; *p* < 0.001), lower body fat, lower diastolic blood pressure, and higher Short Form-36 (SF-36) mental scores. All differences were attenuated by 6 months. No serious adverse events occurred. Total costs were modestly higher in the prehabilitation group (mean, ¥84 843 vs ¥78 051), a difference attributable almost entirely to the prehabilitation programme itself; because the incremental effect on weight loss at 12 months was not statistically significant, a meaningful incremental cost-effectiveness ratio could not be estimated.

**Conclusions and relevance:**

A 6-week multimodal prehabilitation programme accelerated early postoperative weight loss and improved short-term functional outcomes but did not improve total weight loss at 12 months. Because prehabilitation added cost without a demonstrable difference in 12-month weight loss, a cost-effectiveness advantage could not be established. The dominant metabolic effects of bariatric surgery appear to override the incremental gains of preoperative conditioning over time.

## LAY ABSTRACT

People who are severely overweight are often offered weight-loss (bariatric) surgery. Some hospitals now add a short “prehabilitation” programme of exercise, diet advice, and psychological support before surgery to help patients prepare. We compared 60 patients who took part in a 6-week prehabilitation programme with 60 similar patients who received usual preparation, and we followed them for 1 year. Patients who prehabilitated lost more weight and felt better 3 months after surgery, but by 6 and 12 months the 2 groups had caught up with each other and were essentially the same. The extra programme added cost but did not improve weight loss at 1 year, so we found no sign that it saves money or should be recommended on cost grounds. Longer studies are needed to see whether preparing for surgery in this way brings lasting benefit.

Obesity affects more than 1 billion people worldwide and continues to rise at an alarming pace (1). In China alone, over 16% of adults now meet the diagnostic threshold (2). Metabolic and bariatric surgery remains the most effective long-term treatment for severe obesity. It produces sustained weight loss and durable improvements in cardiometabolic health (3, 4). Procedure volumes have grown rapidly across the globe (5). Yet substantial variability persists in postoperative weight loss trajectories, and preoperative factors are among the strongest predictors of outcomes (6). This variability has prompted growing interest in strategies that might optimize patients before they reach the operating room.

Prehabilitation is one such strategy. It refers to a structured, multimodal intervention delivered before surgery to enhance functional capacity and improve postoperative recovery (7). A typical programme integrates 3 pillars: supervised exercise training, nutritional optimization, and psychological support (8). The strongest evidence comes from colorectal cancer surgery. Randomized trials have shown that multimodal prehabilitation improves walking capacity, reduces complications, and shortens hospital stay (9, 10). A recent international multicentre trial reported a 53% reduction in severe complications among prehabilitated patients undergoing colorectal resection (11). These findings have generated considerable momentum. Whether similar benefits extend to the bariatric population, however, remains uncertain. Only 1 pilot trial protocol has specifically examined prehabilitation before bariatric surgery, and its authors concluded that the evidence in this setting is scarce (12).

The economic dimension is equally underexplored. A systematic review of prehabilitation cost-effectiveness identified favourable cost profiles across several surgical populations, yet no study has evaluated costs specifically in bariatric surgery patients (13). One secondary analysis of a prehabilitation trial for major abdominal surgery demonstrated modest programme costs without increasing total expenditures (14). Bariatric surgery itself is cost-saving over a lifetime horizon (15). Whether adding a prehabilitation programme preserves that economic advantage or introduces costs that outweigh the clinical benefit is unknown.

Answering these questions ideally requires a randomized controlled trial. However, practical and logistical constraints can make randomization difficult when a new care pathway is being introduced alongside existing standard practice. Observational studies using propensity score matching offer a rigorous alternative for estimating treatment effects in such circumstances (16, 17). By modelling the probability of treatment assignment conditional on measured baseline covariates and matching treated patients to comparable controls, propensity score matching creates a balanced comparison that reduces confounding bias and strengthens causal inference from nonrandomized data (16, 17).

The purpose of this study was to evaluate the effectiveness and cost-effectiveness of a 6-week structured multimodal prehabilitation programme compared with standard preintervention education in obese patients undergoing metabolic and bariatric surgery, using a retrospective cohort design with propensity score matching. Total weight loss at 12 months served as the primary outcome. We hypothesized that prehabilitation would produce greater total weight loss at 12 months, improve secondary clinical and patient-reported outcomes across the follow-up period, and represent a cost-effective use of healthcare resources relative to standard care.

## METHODS

### Study design and setting

This was a retrospective cohort study comparing multimodal prehabilitation with standard preintervention education in obese patients undergoing primary metabolic and bariatric surgery. All data were extracted from the electronic health records (EHRs) of Shanghai Tenth People’s Hospital linked with Joymotion metadata supported by Shanghai Fudong Medical Management Co., Ltd. (Shanghai, China). The study used a propensity score matched cohort design. Sixty consecutive patients who enrolled in a newly implemented prehabilitation programme formed the treatment group. From a concurrent pool of 254 eligible patients who underwent bariatric surgery with standard preintervention education at the same hospital during the same period, 60 were selected as matched controls through propensity score matching. The study was conducted between January 2022 and January 2025. This study was approved by the Ethics Committee of Shanghai Tenth People’s Hospital (Approval No. SHSY-IEC-6.0/25K117/P01). The study was conducted in conformity with the Declaration of Helsinki (1975, revised 2013). For participants aged 16 to 17 years, additional written parental or guardian consent was obtained.

### Participants

Eligibility required age 16 to 65 years, BMI at or above 27.5 kg/m^2^ with at least 1 obesity-related comorbidity or BMI at or above 32.5 kg/m^2^ regardless of comorbidity status, and being scheduled for primary Roux-en-Y gastric bypass (RYGB) or sleeve gastrectomy (SG).

The inclusion criteria are: age ≥ 16 years, BMI ≥ 27.5 kg/m^2^ with at least 1 obesity-related comorbidity or BMI ≥ 32.5 kg/m^2^ regardless of comorbidity status (in accordance with the Chinese Society for Metabolic and Bariatric Surgery guidelines for Asian populations), and scheduled for primary RYGB or SG.

Patients were excluded if they had undergone prior bariatric or major upper gastrointestinal surgery; had a planned revisional bariatric procedure; were classified as ASA ≥ IV; had uncontrolled cardiac arrhythmia (including atrial fibrillation with rapid ventricular response) or unstable angina; had severe cardiopulmonary disease contraindicating supervised exercise (NYHA class III–IV heart failure or oxygen-dependent COPD); had severe anaemia (haemoglobin < 10 g/dL); had active systemic infection or acute inflammation (CRP > 20 mg/L at screening); met DSM-5 criteria for active substance use disorder; had uncontrolled psychiatric illness precluding safe participation and informed consent; had a musculoskeletal or neurological condition preventing participation in the exercise component; were pregnant or planning pregnancy within 12 months; had end-stage renal disease requiring dialysis; or had active malignancy under treatment. Participants aged 16–17 years required additional written parental or guardian consent.

### Allocation

The prehabilitation programme was introduced as a new clinical care pathway at Shanghai Tenth People’s Hospital beginning in November 2021. Consecutive patients meeting the eligibility criteria and scheduled for primary metabolic/bariatric surgery were offered enrolment in the prehabilitation programme on the basis of the following pragmatic criteria: (*i*) surgical scheduling that allowed a minimum of 8 weeks between the initial consultation and the planned operative date, thereby permitting completion of the prehabilitation programme; (*ii*) geographic residence and logistical circumstances permitting regular attendance at supervised exercise and counselling sessions at the hospital facility; and (*iii*) patient willingness to participate in the structured programme. Patients who could not fulfil these conditions, owing to scheduling constraints, travel distance, employment commitments, or personal preference, received the hospital’s standard preintervention education and served as the concurrent control group.

The type of bariatric surgery (RYGB or SG) was determined independently of group assignment by the multidisciplinary bariatric team based on individual patient anatomy, comorbidity profile, and shared decision-making with the patient, following clinical guidelines. All surgical procedures were performed by the same surgical team using standardized operative techniques and perioperative protocols, regardless of whether the patient had received prehabilitation or standard care. Postoperative management, including dietary advancement, follow-up scheduling, and activity recommendations, was identical for both groups. A total of 120 patients were enrolled during the study period (60 prehabilitation, 60 standard care), with equivalent distributions of surgery type within each group (30 RYGB and 30 SG per group).

## INTERVENTIONS

### Prehabilitation group

Patients assigned to the prehabilitation group completed a 6-week structured, multimodal programme prior to bariatric surgery. The programme integrated 3 pillars: supervised exercise training, nutritional counselling, and psychological support. It was grounded in the trimodal prehabilitation framework developed by Carli and colleagues and adapted for the bariatric population in accordance with the AACE/TOS/ASMBS clinical practice guidelines and the ERAS Society recommendations for perioperative bariatric care (18–22). After surgery, patients in this group received the same postoperative care as the control group (see detailed prehabilitation programme in Supplementary materials).

### Standard preintervention education group

Patients in the control group received standard preintervention education in accordance with the institutional bariatric surgery protocol. This protocol reflected the multidisciplinary preoperative preparation recommended by the AACE/TOS/ASMBS clinical practice guidelines and the Enhanced Recovery After Surgery Society bariatric recommendations (20–22). The programme consisted of 2 structured preoperative counselling sessions delivered within the 6 weeks preceding surgery. No supervised exercise, formal behavioural therapy, or extended lifestyle intervention was provided to the group (see detailed standard preintervention education in Supplementary materials).

### Propensity score matching

Because treatment assignment was not randomized, propensity score matching was used to create a balanced comparison between the prehabilitation and standard care groups and to reduce confounding bias due to differences in baseline characteristics (18). The propensity score was defined as the conditional probability of receiving prehabilitation given the observed baseline covariates, and was estimated using a multivariable logistic regression model (16, 23).

### Propensity score model

The dependent variable in the logistic regression was group assignment, coded as 1 for prehabilitation and 0 for standard care. Covariates were selected *a priori* on clinical and theoretical grounds rather than by data-driven or stepwise procedures. Following established recommendations, we included variables considered to be prognostic for the outcome (postoperative weight loss) or associated with both treatment assignment and outcome, because including outcome-related covariates reduces bias and improves the precision of the estimated treatment effect (17, 33). On this basis, the following 31 baseline covariates were entered simultaneously as independent variables: age, sex, BMI, type of bariatric surgery, education level, insurance type, current smoking status, current alcohol use, C-reactive protein (CRP) level, albumin level, haemoglobin level, presence of hypertension, presence of cardiovascular disease, presence of obstructive sleep apnoea, presence of gastroesophageal reflux disease, bodyweight, SBP, DBP, body fat percentage, waist circumference, fasting glucose, total cholesterol, LDL cholesterol, HDL cholesterol, triglycerides, 6MWT distance, grip strength, SF-36 physical component score, SF-36 mental component score, HADS anxiety score, and HADS depression score.

We recognize that the number of covariates in the propensity score model is large relative to the number of patients treated (60), and that a widely cited rule of thumb recommends at least around 10 events per variable for stable logistic regression (45, 46). That rule, however, was developed for logistic models whose individual coefficients are the object of inference or prediction. Here the logistic regression is used only to estimate a propensity score, a scalar balancing score whose sole purpose is to reproduce covariate balance between groups, not to yield interpretable coefficients or to support causal inference (16, 17). In this balancing role a richer, non-parsimonious covariate set is acceptable and often preferable, and simulation work shows that propensity score approaches are less biased and more precise than outcome logistic regression when events are few relative to the number of confounders (33, 47, 48). We nonetheless took several steps to guard against an unstable or overfitted score. The score was estimated in the full standard-care candidate pool (254 patients) rather than in the matched sample; we verified adequate common support and overlap of the estimated propensity score distributions before matching; and, most importantly, we judged the model by whether it achieved its intended purpose – covariate balance – which it did, with every absolute standardized mean difference below 0.1 and all variance ratios within the 0.8–1.25 range after matching. We agree that adequate balance is not, by itself, proof that the logistic model is correctly specified; we therefore additionally applied covariate adjustment within the matched sample (the linear mixed-effects model described below), which provides a doubly robust estimate that remains consistent if either the propensity score model or the outcome model is correctly specified (37, 38).

### Matching procedure

One-to-one nearest-neighbour matching without replacement was performed on the logit of the propensity score. A caliper width of 0.2 standard deviations of the logit of the propensity score was used, as this has been shown through simulation to eliminate approximately 98% of bias in the estimated treatment effect while maintaining adequate precision (24). Each prehabilitation patient was matched to the single standard care patient with the closest propensity score within the caliper. If no standard care patient fell within the caliper for a given prehabilitation patient, that prehabilitation patient would remain unmatched. In this study, all 60 prehabilitation patients were successfully matched.

### Balance assessment

Covariate balance between the matched groups was assessed using the absolute standardized mean difference for each baseline variable. An absolute SMD below 0.1 was considered to indicate negligible imbalance, consistent with established guidelines (25–29). The distribution of propensity scores was visually inspected in both groups before and after matching to confirm adequate overlap, supporting the positivity assumption. Variance ratios for continuous covariates were examined, with values between 0.8 and 1.25 considered acceptable. The balance assessment for all covariates in the matched cohort is presented in Table I.

**Table I:** Baseline characteristics of the intention-to-treat population

Characteristic	Prehabilitation (*n* = 60)	Standard care (*n* = 60)	*p*-value
Demographics			
Age, years, mean (SD)	32.6 (5.0)	32.6 (5.5)	0.94
BMI, kg/m², mean (SD)	38.1 (2.8)	38.2 (3.5)	0.76
Bodyweight, kg, median (IQR)	116.9 (105.7–123.8)	115.5 (106.1–123.0)	0.47
Male sex, *n* (%)	41 (68.3)	38 (63.3)	0.70
Education ≥ high school, *n* (%)	40 (66.7)	39 (65.0)	1.00
Insurance type, *n* (%)			0.53
Government	44 (73.3)	46 (76.7)	
Commercial	11 (18.3)	7 (11.7)	
Self-financed	5 (8.3)	7 (11.7)	
Lifestyle factors			
Current smoker, *n* (%)	13 (21.7)	9 (15.0)	0.48
Current alcohol use, *n* (%)	11 (18.3)	10 (16.7)	1.00
Laboratory values			
CRP, mg/L, mean (SD)	9.8 (2.1)	9.8 (1.5)	0.98
Albumin, g/dL, mean (SD)	4.3 (0.6)	4.2 (0.5)	0.75
Haemoglobin, g/dL, mean (SD)	13.8 (1.0)	13.7 (1.1)	0.69
Medical history			
Hypertension, *n* (%)	2 (3.3)	4 (6.7)	0.68
Cardiovascular disease, *n* (%)	2 (3.3)	3 (5.0)	1.00
Atrial fibrillation, *n* (%)	0 (0.0)	0 (0.0)	1.00
Obstructive sleep apnoea, *n* (%)	9 (15.0)	7 (11.7)	0.79
H. pylori infection, *n* (%)	1 (1.7)	1 (1.7)	1.00
Hepatitis, *n* (%)	1 (1.7)	1 (1.7)	1.00
GERD, *n* (%)	1 (1.7)	2 (3.3)	1.00
Surgical characteristics			
Type of bariatric surgery, *n* (%)			1.00
RYGB	30 (50.0)	30 (50.0)	
Sleeve gastrectomy	30 (50.0)	30 (50.0)	
Vital signs and anthropometrics			
SBP, mmHg, mean (SD)	118.8 (7.2)	117.9 (7.4)	0.50
DBP, mmHg, mean (SD)	77.8 (6.6)	77.5 (9.0)	0.84
Body fat, %, median (IQR)	37.0 (35.0–40.2)	38.0 (34.8–42.0)	0.80
Waist circumference, cm, median (IQR)	129.1 (116.0–138.3)	125.8 (118.4–135.6)	0.67
Metabolic parameters			
Fasting glucose, mmol/L, mean (SD)	109.0 (13.6)	108.3 (16.1)	0.81
Total cholesterol, mmol/L, mean (SD)	195.2 (12.6)	197.2 (11.7)	0.38
LDL cholesterol, mmol/L, mean (SD)	114.0 (10.6)	115.1 (10.3)	0.58
HDL cholesterol, mmol/L, mean (SD)	55.9 (4.4)	56.8 (5.2)	0.30
Triglycerides, mmol/L, mean (SD)	128.0 (15.9)	131.2 (17.9)	0.32
Functional measures			
6MWT distance, m, mean (SD)	308.3 (59.9)	310.2 (52.5)	0.85
Grip strength, kg force, mean (SD)	35.0 (3.5)	34.7 (3.7)	0.65
Patient-reported outcomes			
SF-36 Physical, mean (SD)	49.8 (6.0)	51.0 (7.5)	0.33
SF-36 Mental, mean (SD)	50.9 (7.1)	51.1 (7.0)	0.50
HADS Anxiety score, mean (SD)	15.3 (2.8)	15.2 (2.7)	0.84
HADS Depression score, mean (SD)	15.0 (2.6)	14.5 (2.7)	0.29

BMI: body mass index; CRP: C-reactive protein; GERD: gastroesophageal reflux disease; RYGB: Roux-en-Y gastric bypass; SBP: systolic blood pressure; DBP: diastolic blood pressure; 6MWT: 6-minute walk test; SF-36: Short Form-36; HADS: Hospital Anxiety and Depression Scale. Continuous variables are presented as mean (SD) or median (IQR) based on distribution. Categorical variables are presented as *n* (%). *P*-values were calculated using an independent *t*-test or Mann–Whitney *U*-test for continuous variables and χ^2^ or Fisher’s exact test for categorical variables.

After matching, all absolute SMDs were below 0.1, indicating that the propensity score matching procedure achieved adequate balance across the 31 baseline covariates included in the model. The matched cohort of 120 patients therefore served as the primary analytic sample for all subsequent.

### Outcomes

The primary outcome was total weight loss (TWL) measured in kilograms at 12 months post-surgery. TWL was calculated as baseline bodyweight minus bodyweight at 12 months. Secondary efficacy outcomes included percentage excess weight loss (%EWL, calculated using an ideal BMI of 25 kg/m^2^), BMI, body fat percentage, waist circumference, systolic and diastolic blood pressure (SBP, DBP), fasting glucose, lipid profile components (total cholesterol, low-density lipoprotein [LDL], high-density lipoprotein [HDL], triglycerides), 6-minute walk test (6MWT) distance, handgrip strength, Short Form-36 (SF-36) physical and mental component scores, and Hospital Anxiety and Depression Scale (HADS) anxiety and depression subscores (30). The HADS is a validated 14-item self-report instrument comprising separate 7-item anxiety and depression subscales, each scored from 0 to 21, with higher scores indicating greater symptom burden (30). All secondary outcomes were measured at 3, 6, and 12 months post-surgery.

Safety outcomes included any serious adverse event (SAE), specific surgical or device-related complications (gastrointestinal bleeding, device migration, gastrointestinal obstruction/perforation, hepatic abscess, pancreatitis), number of hospitalizations, number of emergency room (ER) visits related to the intervention, and additional procedures (e.g., endoscopy for complications). Safety was assessed at 3, 6, and 12 months.

For the cost-effectiveness analysis, costs were classified into 3 categories: (*i*) direct medical costs, comprising the intervention (surgical procedure plus device procurement), prehabilitation programme costs, follow-up visit costs, and complication management costs; (*ii*) direct non-medical costs, including transportation to the hospital and dietary programme costs borne out of pocket; and (*iii*) indirect costs, comprising lost wages for participants and lost wages for family members. All costs were assessed at 3 months post-surgery and expressed in Chinese Yuan (¥). Exercise adherence (average weekly exercise minutes, weeks meeting the ≥ 150 min/week recommendation, and physical activity log completion) and dietary adherence (average daily caloric intake, days achieving calorie goals of ≤ 1200–1500 kcal/day, days with sufficient protein intake of ≥ 60–80 g/day, and attendance at dietary counselling sessions) were assessed at 12 months.

## STATISTICAL ANALYSIS

### Descriptive analysis

Baseline characteristics of the matched cohort were summarized as mean with standard deviation for normally distributed continuous variables, median with interquartile range for non-normally distributed continuous variables, and frequency with percentage for categorical variables. Normality was assessed using the Shapiro–Wilk test. Between-group balance was reported using absolute standardized mean differences rather than *p*-values, as *p*-values are not appropriate for assessing balance in matched samples.

### Primary analysis: unadjusted comparisons

Unadjusted between-group differences in outcomes at 3, 6, and 12 months were estimated using independent-samples *t*-tests. Mean differences with 95% confidence intervals (CIs) were calculated using the Welch approximation to account for potential unequal variances. These unadjusted analyses served as the initial assessment of treatment effects across the ITT population.

### Primary analysis: adjusted linear mixed-effects model

Although propensity score matching balanced measured baseline covariates between groups, it does not by itself account for the correlation among repeated measurements obtained from the same patient at 3, 6, and 12 months. We therefore used a linear mixed-effects model (LMM) as the primary adjusted analysis for 2 complementary reasons. First, the LMM is designed for longitudinal (repeated-measurement) data with a continuous outcome and models the within-patient correlation across time points, which a matched cross-sectional comparison cannot do (39, 40). Second, combining matching with additional regression adjustment for a small set of strong prognostic covariates provides a doubly robust estimate: the treatment effect remains consistent if either the matching (propensity score) model or the outcome (regression) model is correctly specified, and this combination reduces any residual imbalance that may persist after matching while improving precision (37, 38). The adjustment covariates in the LMM (age, sex, baseline BMI, and surgery type) are a deliberately parsimonious subset of well-established prognostic factors for post-bariatric weight loss; their inclusion in both the propensity score model and the outcome model is consistent with recommended double-adjustment practice and does not constitute circular reasoning, because the 2 models serve distinct roles (constructing the matched sample vs estimating the adjusted longitudinal effect) (37, 38).

The model was specified as follows: the dependent variable was the outcome of interest (e.g., TWL, BMI, %EWL, or any secondary outcome); the primary independent variable was prehabilitation group assignment (prehabilitation = 1 vs standard care = 0); fixed effects included group, time (3, 6, and 12 months, treated as categorical), the group × time interaction, age, sex, baseline BMI, and type of bariatric surgery; and a random intercept was included for each patient. The patient-level random intercept represents the correlation among repeated measurements taken from the same individual – that is, the tendency of a given patient’s outcomes to cluster around that patient’s own mean level – rather than any claim to recover unmeasured confounders; unmeasured variables are not, and cannot be, estimated by the model. The group coefficient (β) represents the adjusted mean difference between prehabilitation and standard care, with positive values indicating higher values in the prehabilitation group. Time-stratified models adjusting for age, sex, baseline BMI, and surgery type were additionally fitted at each time point (3, 6, and 12 months separately) using ordinary least-squares regression to facilitate interpretation of time-specific effects. Model parameters were estimated using restricted maximum likelihood (REML). The LMM accommodates missing data under the missing-at-random (MAR) assumption by using all available observations without requiring imputation. Model assumptions were assessed graphically and statistically: normality of the conditional residuals was examined using quantile–quantile plots and the Shapiro–Wilk test, homoscedasticity was assessed from plots of residuals against fitted values, and the normality of the random intercepts was checked using quantile–quantile plots of the empirical best linear unbiased predictors. No material departures were observed, and linear mixed models are additionally known to be robust to mild violations of these distributional assumptions (34–36). Diagnostic details are provided in the Supplementary materials.

*Missing data.* Baseline data were complete for all 120 participants. During follow-up, missing outcome data arose only from participants who did not attend a scheduled visit, in whom all outcomes for that visit were missing simultaneously (there were no intermittently missing individual variables among attending participants). Missing outcome data at 12 months amounted to 5.0% (3/60) in the prehabilitation group and 8.3% (5/60) in the standard care group. The linear mixed-effects model accommodates incomplete follow-up under the missing-at-random assumption by using all available observations across time points without imputation.

### Cost-effectiveness analysis

The economic analysis adopted a societal perspective and incorporated direct medical, direct non-medical, and indirect costs. Total costs at 3 months were compared between groups using the Mann–Whitney *U*-test and are reported descriptively as mean (SD). We had prespecified an incremental cost-effectiveness ratio (ICER), defined as the incremental cost divided by the incremental effect (the between-group difference in TWL at 12 months), with uncertainty to be characterized by nonparametric bootstrapping. In the bootstrap, complete patient-level records were resampled with replacement so that each patient’s cost and effect values were kept together as a paired unit, preserving the within-patient association between cost and effect; the incremental cost and incremental effect were then recomputed in each of 5,000 resamples to generate the joint distribution displayed on the cost-effectiveness plane. However, because the incremental effect on TWL at 12 months was not statistically significant (and therefore not distinguishable from zero), a point-estimate ICER is uninterpretable: the ratio is unstable when its denominator approaches zero, its confidence limits span multiple quadrants of the cost-effectiveness plane, and the resulting value cannot be meaningfully compared with a willingness-to-pay threshold (43, 44). We therefore do not report a point-estimate ICER and instead present the cost components descriptively alongside the cost-effectiveness plane, which conveys the underlying uncertainty.

### Sensitivity and subgroup analyses

Several prespecified sensitivity and subgroup analyses were conducted to assess the robustness of findings. First, a per-protocol analysis was performed restricting the sample to patients with complete 12-month follow-up data (prehabilitation: *n* = 57; standard care: *n* = 55) to evaluate whether the results were sensitive to loss to follow-up. Second, a subgroup analysis stratified by type of bariatric surgery (RYGB vs SG) was conducted to assess whether treatment effects differed by surgical procedure. Interaction *p*-values were obtained from linear regression models including a group × surgery type interaction term. Third, adherence to exercise and dietary recommendations was compared between groups at 12 months to evaluate potential behavioural mediators. All statistical tests were 2-sided, and *p* < 0.05 was considered statistically significant. Analyses were performed using Python 3.12 (https://www.python.org/) with pandas, SciPy, and statsmodels packages.

## RESULTS

### Participant selection and flow

**Fig. 1:**
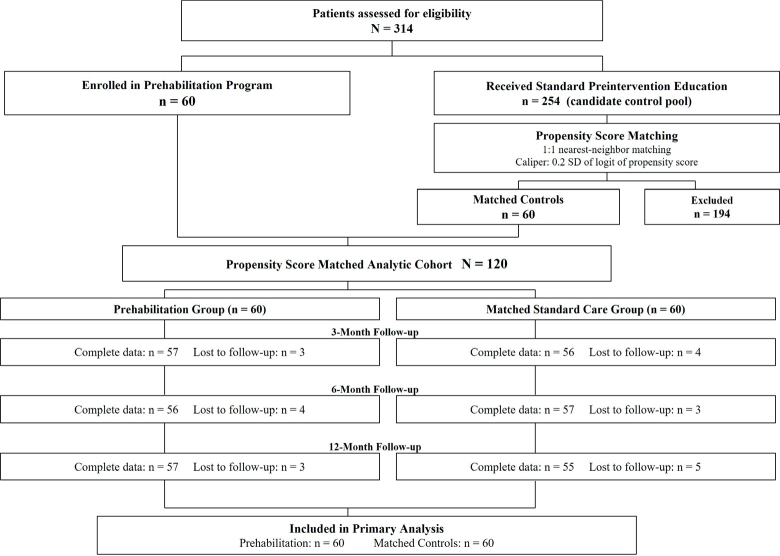
Flow diagram of the study. PSM: propensity score matching; SD: standard deviation. The 194 unmatched standard care patients were excluded from the primary analysis but included in a supplementary sensitivity analysis on the full sample (*n* = 314). All 60 prehabilitation patients were successfully matched. No patients crossed over, withdrew consent, or died during follow-up.

During the study period, 60 patients enrolled in the prehabilitation programme. Concurrently, 254 patients meeting the same eligibility criteria underwent bariatric surgery with standard preintervention education. From these 254 candidates, 60 were selected as matched controls through 1:1 propensity score matching, yielding a final analytic cohort of 120 patients. The flow diagram of the study is shown in Fig. 1.

The remaining 120 eligible patients were assigned to the prehabilitation group (*n* = 60) or the standard preintervention education group (*n* = 60). Within each group, 30 patients underwent RYGB and 30 underwent SG. At 3 months, complete data were available for 57 prehabilitation participants (95.0%) and 56 control participants (93.3%). At 6 months, 56 prehabilitation participants (93.3%) and 57 control participants (95.0%) had complete data. At 12 months, 57 prehabilitation participants (95.0%) and 55 control participants (91.7%) completed follow-up. Overall retention at 12 months was 93.3% (112/120). No participants crossed over between groups, withdrew consent, or died during the study period.

### Baseline characteristics

Baseline characteristics of the matched (intention-to-treat (ITT)) cohort are presented in Table I. After matching, the prehabilitation and standard care groups were well balanced on all 31 covariates, with every absolute standardized mean difference (SMD) below the prespecified threshold of 0.1. Because balance rather than statistical comparison is the relevant criterion in a matched sample, we direct the reader to Table I for the full covariate distribution and report no between-group significance tests for baseline variables. Baseline data were complete for all 120 participants.

### Follow-up

All participants were followed for up to 12 months post-surgery, with assessments scheduled at 3, 6, and 12 months. The follow-up period was identical for both treatment strategies. Loss to follow-up was modest and occurred at different rates across time points: at 3 months, 3 prehabilitation participants (5.0%) and 4 control participants (6.7%) had missing outcome data; at 6 months, 4 (6.7%) and 3 (5.0%); and at 12 months, 3 (5.0%) and 5 (8.3%), respectively. All missing data resulted from failure to attend scheduled follow-up visits. No participants experienced protocol deviations requiring exclusion from the per-protocol analysis beyond those lost to follow-up.

### Primary outcome

For the primary outcome, total weight loss (TWL) at 12 months did not differ significantly between groups: mean TWL was 23.7 (SD 5.1) kg with prehabilitation and 23.4 (SD 2.7) kg with standard care, a difference of 0.3 kg (95% CI, −1.3 to 1.8; *p* = 0.75) that was neither statistically significant nor clinically meaningful (Table II).

**Table II:** Unadjusted changes in outcomes at months 3, 6, and 12 (ITT population)

Outcome	Month	Prehabilitation, mean (SD)	Standard care, mean (SD)	Difference (95% CI)	*p*-value	No. (Preh/Std)
Total weight loss, kg*	3	23.7 (4.2)	20.7 (1.4)	2.9 (1.8 to 4.1)	< 0.001	57 / 56
	6	24.0 (5.0)	23.8 (1.4)	0.2 (−1.2 to 1.6)	0.76	56 / 57
	12	23.7 (5.1)	23.4 (2.7)	0.3 (−1.3 to 1.8)	0.75	57 / 55
%EWL	3	57.1 (11.5)	51.4 (12.8)	5.7 (1.2 to 10.2)	0.01	57 / 56
	6	58.0 (12.9)	58.9 (14.2)	−0.9 (−5.9 to 4.1)	0.73	56 / 57
	12	57.1 (12.9)	58.1 (12.5)	−1.0 (−5.7 to 3.7)	0.69	57 / 55
BMI, kg/m²	3	30.2 (2.4)	31.3 (3.2)	−1.1 (−2.1 to −0.0)	0.04	57 / 56
	6	30.1 (2.4)	30.2 (3.0)	−0.1 (−1.1 to 0.9)	0.79	56 / 57
	12	30.2 (2.3)	30.3 (3.0)	−0.1 (−1.1 to 0.9)	0.87	57 / 55
Bodyweight, kg	3	91.3 (8.5)	93.4 (9.6)	−2.1 (−5.4 to 1.3)	0.22	57 / 56
	6	90.6 (8.0)	90.4 (9.5)	0.2 (−3.1 to 3.4)	0.92	56 / 57
	12	90.9 (8.0)	90.4 (8.7)	0.5 (−2.6 to 3.6)	0.76	57 / 55
Body fat, %	3	30.2 (3.7)	32.2 (4.1)	−1.9 (−3.4 to −0.5)	0.01	57 / 56
	6	30.0 (2.8)	30.3 (3.7)	−0.3 (−1.5 to 0.9)	0.61	56 / 57
	12	30.3 (3.0)	30.3 (3.7)	−0.0 (−1.3 to 1.2)	0.94	57 / 55
SBP, mmHg	3	116.2 (8.0)	117.8 (6.6)	−1.6 (−4.3 to 1.1)	0.25	57 / 56
	6	117.9 (10.1)	117.6 (9.4)	0.4 (−3.2 to 4.0)	0.84	56 / 57
	12	116.2 (10.5)	116.6 (11.7)	−0.3 (−4.5 to 3.8)	0.88	57 / 55
DBP, mmHg	3	72.1 (5.8)	74.8 (6.9)	−2.7 (−5.0 to −0.3)	0.03	57 / 56
	6	79.2 (7.7)	79.9 (8.6)	−0.7 (−3.7 to 2.3)	0.63	56 / 57
	12	79.1 (9.0)	78.5 (8.6)	0.6 (−2.6 to 3.9)	0.71	57 / 55
Fasting glucose, mmol/L	3	96.7 (13.8)	99.9 (16.4)	−3.3 (−8.9 to 2.3)	0.26	57 / 56
	6	96.1 (14.0)	96.8 (12.6)	−0.6 (−5.5 to 4.3)	0.81	56 / 57
	12	87.1 (13.2)	86.7 (12.5)	0.5 (−4.3 to 5.2)	0.85	57 / 55
6MWT distance, m	3	389.5 (65.3)	372.8 (57.2)	16.7 (−5.9 to 39.4)	0.15	57 / 56
	6	379.2 (63.9)	380.1 (52.1)	−0.9 (−22.4 to 20.6)	0.94	56 / 57
	12	384.1 (60.3)	381.6 (46.5)	2.5 (−17.4 to 22.4)	0.81	57 / 55
Grip strength, kg force	3	38.0 (3.7)	36.6 (3.7)	1.4 (0.0 to 2.8)	0.04	57 / 56
	6	38.0 (3.9)	37.4 (3.8)	0.5 (−0.9 to 2.0)	0.46	56 / 57
	12	35.8 (4.0)	35.5 (3.8)	0.3 (−1.1 to 1.8)	0.67	57 / 55
SF-36 Physical	3	65.6 (6.5)	63.2 (8.1)	2.4 (−0.3 to 5.1)	0.09	57 / 56
	6	65.5 (7.8)	65.8 (7.7)	−0.3 (−3.2 to 2.6)	0.83	56 / 57
	12	70.4 (7.9)	70.5 (7.7)	−0.1 (−3.0 to 2.8)	0.92	57 / 55
SF-36 Mental	3	66.0 (7.7)	63.1 (7.3)	2.9 (0.2 to 5.7)	0.04	57 / 56
	6	65.7 (8.2)	65.1 (7.8)	0.6 (−2.4 to 3.5)	0.71	56 / 57
	12	70.8 (8.4)	70.1 (8.0)	0.7 (−2.3 to 3.8)	0.64	57 / 55
HADS Anxiety	3	12.4 (3.1)	13.1 (2.9)	−0.8 (−1.9 to 0.3)	0.18	57 / 56
	6	12.5 (2.7)	12.4 (2.7)	0.1 (−0.9 to 1.1)	0.85	56 / 57
	12	10.3 (2.8)	10.5 (2.7)	−0.2 (−1.2 to 0.8)	0.71	57 / 55
HADS Depression	3	11.7 (2.8)	12.4 (3.1)	−0.7 (−1.8 to 0.4)	0.23	57 / 56
	6	12.2 (2.0)	12.3 (2.5)	−0.1 (−1.0 to 0.7)	0.75	56 / 57
	12	10.3 (2.2)	10.1 (2.2)	0.2 (−0.6 to 1.0)	0.68	57 / 55

* Primary outcome. Values are mean (SD). Difference is prehabilitation minus standard care for weight-related outcomes. 95% CI computed using Welch t-test. %EWL: percentage excess weight loss; BMI: body mass index; SBP: systolic blood pressure; DBP: diastolic blood pressure; 6MWT: 6-minute walk test; SF-36: Short Form-36; HADS: Hospital Anxiety and Depression Scale. The “No. (Preh/Std)” column reports the number of participants with available data at each time point in the prehabilitation and standard care groups, respectively (e.g., HADS, SF-36, and all other outcomes were assessed in these participants).

**Fig. 2:**
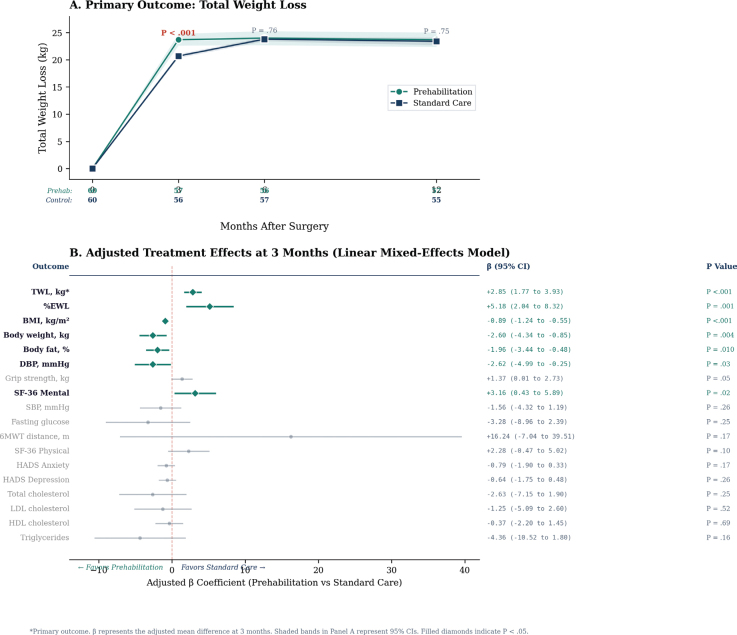
Primary outcome. Panel A shows the primary outcome trajectory as a line plot with 95% confidence bands. The prehabilitation group (teal circles) and standard care group (navy squares) both start at zero and diverge sharply at 3 months (23.7 vs 20.7 kg, *p* < 0.001), then converge by 6 and 12 months. The shaded bands are narrow for the control group (reflecting the smaller SD of 1.4 kg at 3 months) and wider for prehabilitation (SD 4.2 kg). Sample sizes for each group at each timepoint are displayed below the x-axis. Panel B is a forest plot of the adjusted treatment effects from the linear mixed-effects model at 3 months for all 18 outcomes. Statistically significant results are displayed as filled teal diamonds with bold labels; non-significant results appear as faded grey circles. The dashed red vertical line marks zero (no effect). Each row shows the outcome name on the left, the point estimate with horizontal confidence interval in the centre, and the numeric beta coefficient, 95% CI, and *p*-value on the right.

A statistically significant early benefit was observed at 3 months. At that time point, mean TWL was 2.9 kg higher with prehabilitation than with standard care (23.7 (SD 4.2) vs 20.7 (SD 1.4) kg; 95% CI, 1.8 to 4.1; *p* < 0.001). This difference was no longer significant at 6 months (0.2 kg; 95% CI, −1.2 to 1.6; *p* = 0.76) or at 12 months. The same early-benefit-then-convergence pattern was seen for percentage excess weight loss (%EWL; 3-month difference, 5.7 percentage points higher with prehabilitation; *p* = 0.01; 12-month *p* = 0.69) and for BMI (3-month difference, 1.1 kg/m^2^ lower with prehabilitation; *p* = 0.04; 12-month *p* = 0.87). Full results for all outcomes at 3, 6, and 12 months are provided in Table II and Fig. 2.

### Secondary outcomes

At 3 months, prehabilitation was associated with statistically significant improvements in 4 secondary outcomes relative to standard care: body fat percentage was 1.9 percentage points lower (30.2% (SD 3.7) vs 32.2% (SD 4.1); *p* = 0.01), diastolic blood pressure (DBP) was 2.7 mmHg lower (72.1 (SD 5.8) vs 74.8 (SD 6.9) mmHg; *p* = 0.03), grip strength was 1.4 kg higher (38.0 (SD 3.7) vs 36.6 (SD 3.7) kg; *p* = 0.04), and the SF-36 mental component score was 2.9 points higher (66.0 (SD 7.7) vs 63.1 (SD 7.3); *p* = 0.04). None of these differences remained statistically significant at 6 or 12 months. No statistically significant between-group difference was observed at any time point for systolic blood pressure, fasting glucose, the lipid profile, waist circumference, 6-minute walk test distance, the SF-36 physical component score, or the HADS anxiety and depression scores (see Table II).

### Adjusted effect estimates

The adjusted linear mixed-effects models (Table III) were consistent with the unadjusted results. For the primary outcome, prehabilitation was associated with a significantly greater TWL at 3 months: the adjusted mean difference was 2.85 kg higher than standard care (95% CI, 1.77 to 3.93; *p* < 0.001). This adjusted difference was small and non-significant at 6 months (0.17 kg; 95% CI, −1.11 to 1.45; *p* = 0.79) and at 12 months (0.17 kg; 95% CI, −1.17 to 1.52; *p* = 0.80). Averaged across the 3 time points, the overall adjusted difference was 2.81 kg (95% CI, 1.62 to 4.01; *p* < 0.001), a value driven by the large 3-month effect.

**Table III:** Adjusted effectiveness of prehabilitation using linear mixed-effects models

Outcome	Time point	β (Group)	95% CI	*p*-value	No. (Preh/Std)
Total weight loss, kg*	3 months	2.85	(1.77 to 3.93)	< 0.001	57 / 56
	6 months	0.17	(−1.11 to 1.45)	0.79	56 / 57
	12 months	0.17	(−1.17 to 1.52)	0.80	57 / 55
	Overall	2.81	(1.62 to 4.01)	< 0.001	60 / 60
%EWL	3 months	5.18	(2.04 to 8.32)	0.001	57 / 56
	6 months	−1.35	(−5.04 to 2.33)	0.47	56 / 57
	12 months	−1.22	(−4.83 to 2.39)	0.51	57 / 55
	Overall	5.05	(1.71 to 8.39)	0.003	60 / 60
BMI, kg/m²	3 months	−0.89	(−1.24 to −0.55)	< 0.001	57 / 56
	6 months	−0.00	(−0.41 to 0.41)	1.00	56 / 57
	12 months	−0.01	(−0.44 to 0.43)	0.97	57 / 55
	Overall	−0.88	(−1.26 to −0.49)	< 0.001	60 / 60
Bodyweight, kg	3 months	−2.60	(−4.34 to −0.85)	0.004	57 / 56
	6 months	−0.14	(−1.85 to 1.56)	0.87	56 / 57
	12 months	0.11	(−1.61 to 1.82)	0.90	57 / 55
	Overall	−2.39	(−4.09 to −0.69)	0.006	60 / 60
Body fat, %	3 months	−1.96	(−3.44 to −0.48)	0.010	57 / 56
	6 months	−0.32	(−1.54 to 0.91)	0.61	56 / 57
	12 months	−0.06	(−1.31 to 1.20)	0.93	57 / 55
	Overall	−1.81	(−3.10 to −0.53)	0.006	60 / 60
SBP, mmHg	3 months	−1.56	(−4.32 to 1.19)	0.26	57 / 56
	6 months	0.25	(−3.35 to 3.86)	0.89	56 / 57
	12 months	−0.38	(−4.55 to 3.78)	0.86	57 / 55
	Overall	−1.87	(−5.38 to 1.64)	0.30	60 / 60
DBP, mmHg	3 months	−2.62	(−4.99 to −0.25)	0.03	57 / 56
	6 months	−0.84	(−3.81 to 2.13)	0.58	56 / 57
	12 months	0.61	(−2.70 to 3.92)	0.72	57 / 55
	Overall	−2.62	(−5.49 to 0.26)	0.07	60 / 60
Fasting glucose, mmol/L	3 months	−3.28	(−8.96 to 2.39)	0.25	57 / 56
	6 months	−0.63	(−5.66 to 4.40)	0.80	56 / 57
	12 months	0.44	(−4.43 to 5.32)	0.86	57 / 55
	Overall	−2.32	(−7.44 to 2.80)	0.37	60 / 60
6MWT distance, m	3 months	16.24	(−7.04 to 39.51)	0.17	57 / 56
	6 months	−0.74	(−22.65 to 21.17)	0.95	56 / 57
	12 months	2.06	(−18.37 to 22.49)	0.84	57 / 55
	Overall	17.57	(−3.46 to 38.60)	0.10	60 / 60
Grip strength, kg force	3 months	1.37	(0.01 to 2.73)	0.05	57 / 56
	6 months	0.54	(−0.88 to 1.96)	0.45	56 / 57
	12 months	0.29	(−1.16 to 1.73)	0.70	57 / 55
	Overall	1.27	(−0.09 to 2.62)	0.07	60 / 60
SF-36 Physical	3 months	2.28	(−0.47 to 5.02)	0.10	57 / 56
	6 months	−0.42	(−3.30 to 2.47)	0.78	56 / 57
	12 months	−0.28	(−3.21 to 2.65)	0.85	57 / 55
	Overall	1.62	(−1.16 to 4.40)	0.25	60 / 60
SF-36 Mental	3 months	3.16	(0.43 to 5.89)	0.02	57 / 56
	6 months	0.62	(−2.30 to 3.54)	0.67	56 / 57
	12 months	0.86	(−2.17 to 3.90)	0.57	57 / 55
	Overall	2.95	(0.20 to 5.70)	0.04	60 / 60
HADS Anxiety	3 months	−0.79	(−1.90 to 0.33)	0.17	57 / 56
	6 months	0.06	(−0.95 to 1.08)	0.90	56 / 57
	12 months	−0.20	(−1.24 to 0.84)	0.70	57 / 55
	Overall	−0.78	(−1.80 to 0.24)	0.13	60 / 60
HADS Depression	3 months	−0.64	(−1.75 to 0.48)	0.26	57 / 56
	6 months	−0.15	(−1.02 to 0.72)	0.73	56 / 57
	12 months	0.19	(−0.64 to 1.01)	0.66	57 / 55
	Overall	−0.70	(−1.64 to 0.24)	0.15	60 / 60

* Primary outcome. The linear mixed-effects model included prehabilitation group as the independent variable with random intercepts for each patient to account for within-individual correlation across repeated measurements. Time-stratified models at months 3, 6, and 12 adjusted for age, sex, baseline BMI, and surgery type. The overall model included group, time, group × time interaction, age, sex, baseline BMI, and surgery type as fixed effects. β coefficient represents the adjusted mean difference between prehabilitation and standard care groups. Positive β indicates higher values in the prehabilitation group. The “No. (Preh/Std)” column reports the number of participants contributing data at each time point (prehabilitation/standard care); the Overall row reflects the full matched cohort (60 / 60), because the mixed-effects model uses all available observations across time points.

The adjusted models confirmed the same early, transient pattern for the secondary outcomes that were significant in the unadjusted analysis, including %EWL, BMI, bodyweight, body fat percentage, DBP, grip strength, and the SF-36 mental component score: each showed a statistically significant difference favouring prehabilitation at 3 months that converged to non-significance by 6 and 12 months (see Table III). No statistically significant adjusted difference was found at any time point for systolic blood pressure, fasting glucose, the lipid profile, waist circumference, 6-minute walk test distance, the SF-36 physical component score, or the HADS scores.

### Cost analysis

Table IV presents the cost comparison at 3 months, with all values expressed as mean (SD) in Chinese Yuan (¥). Total mean (SD) cost was ¥84,843 (5,251) in the prehabilitation group and ¥78,051 (6,002) in the standard care group. The 2 groups had nearly identical surgical (intervention) costs (¥40,302 [518] vs ¥40,323 [515]; *p* = 0.98), follow-up visit costs (¥314 [93] vs ¥309 [84]; *p* = 0.91), direct non-medical costs (transportation and dietary program; subtotal ¥9,834 vs ¥9,798), and indirect costs (lost wages for participants, ¥23,083 [12,624] vs ¥23,127 [14,785], *p* = 0.58; and for family members, ¥4,329 [7,011] vs ¥4,494 [7,343], *p* = 0.97). No complication-management costs were incurred in either group. Consequently, the entire ¥6,792 difference in total cost was due to the prehabilitation programme fee of ¥6,980 per patient, which by design was incurred only by the prehabilitation group and had no within-group variability (SD, 0).

**Table IV:** Costs of the prehabilitation and standard care groups at 3 months (¥, Chinese Yuan)

Cost category	Prehabilitation (*n* = 57)	Standard care (*n* = 56)	*p*-value
Direct medical cost			
Intervention cost	40,302 (518)	40,323 (515)	0.98
Prehabilitation cost	6,980 (0)	0 (0)	—
Follow-up visits	314 (93)	309 (84)	0.91
Complication management	0 (0)	0 (0)	1.00
Subtotal	47,597	40,632	
Direct non-medical cost			
Transportation	1,883 (318)	1,821 (309)	0.40
Dietary programme	7,951 (996)	7,978 (941)	0.94
Subtotal	9,834	9,798	
Indirect cost			
Lost wages (participant)	23,083 (12,624)	23,127 (14,785)	0.58
Lost wages (family member)	4,329 (7,011)	4,494 (7,343)	0.97
Subtotal	27,412	27,621	
Total cost	84,843	78,051	

Values are mean (SD) in Chinese Yuan (¥). *P*-values were calculated using the Mann–Whitney *U*-test. The prehabilitation programme cost was applicable only to the prehabilitation group. Cost data were available for 57 prehabilitation and 56 standard care participants with complete 3-month follow-up. Costs are presented descriptively; because the incremental effect on total weight loss at 12 months was not statistically significant, an incremental cost-effectiveness ratio was not computed (see text). The prehabilitation programme fee was a fixed per-patient charge incurred only by the prehabilitation group and therefore has no within-group variability (SD, 0).

### Cost-effectiveness

**Fig. 3:**
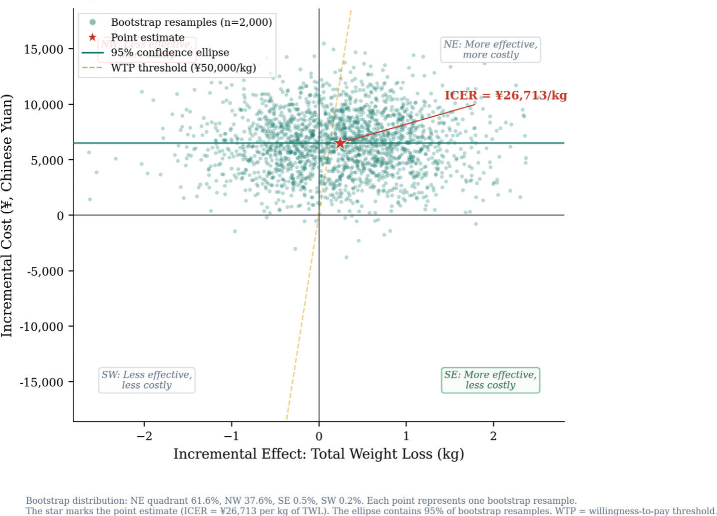
Cost-effectiveness plane. Each of the bootstrap resamples appears as a teal dot, with the point estimate of incremental cost and effect marked at the centre of the cloud, around which a 95% confidence ellipse is drawn. The 4 quadrants are labelled in the corners: NE (more effective, more costly), NW (less effective, more costly), SE (more effective, less costly), and SW (less effective, less costly). Because the incremental effect on total weight loss at 12 months was not statistically significant, the bootstrap cloud straddles the vertical axis of no effect: 61.6% of resamples fell in the NE quadrant and 37.6% in the NW quadrant, indicating substantial uncertainty about whether prehabilitation is more or less effective, while nearly all resamples showed higher cost. Consistent with this uncertainty, no point-estimate incremental cost-effectiveness ratio is reported.

A formal cost-effectiveness analysis does not add interpretable information in this setting, for 2 reasons. First, prehabilitation produced no statistically significant difference in the primary effectiveness outcome (TWL at 12 months) or in any other 12-month outcome, so there is no durable effectiveness gain against which to weigh cost. Second, the only material cost difference – the prehabilitation programme fee – was incurred by every prehabilitation patient and by no control patient; it therefore has no within-group variability and carries no statistical uncertainty, so a comparative test of costs is not meaningful. Taken together, these data provide no evidence that prehabilitation combined with metabolic and bariatric surgery is cost-effective, and on the basis of this study it cannot be recommended as a cost-effective addition to standard care. This does not exclude the possibility that prehabilitation is cost-effective: the statistically significant short-term (3-month) improvements in weight loss and functional and patient-reported outcomes could translate into economic value over a longer horizon, but the present 12-month data cannot demonstrate this. For completeness, and recognizing that it rests on a non-significant effect difference, the cost-effectiveness plane is presented in Fig. 3 for illustrative purposes only, to convey the joint uncertainty in incremental cost and effect.

### Safety

No serious adverse events were observed in either group during the entire 12-month follow-up period. No specific surgical or device-related complications (gastrointestinal bleeding, device migration, gastrointestinal obstruction/perforation, hepatic abscess, or pancreatitis) were reported. All participants in both groups had exactly 1 hospitalization (for the index surgery itself) and zero ER visits related to the intervention at each time point. No additional procedures (e.g., endoscopy for complications) were required. The safety profile was identical between the prehabilitation and control groups (Table SI).

### Additional analyses

*Exercise and dietary adherence (Tables SII and SIII).* At 12 months, the prehabilitation group reported significantly greater average weekly exercise (136.5 (15.9) vs 99.6 (9.2) min; *p* < 0.001) and higher physical activity log completion (126.8 [15.8] vs 105.3 [28.7] of a possible 156 entries; *p* < 0.001). However, the number of weeks meeting the recommended ≥ 150 min per week did not differ significantly (19.2 [11.2] vs 15.9 [4.8]; *p* = 0.28). With regard to dietary adherence, the prehabilitation group had a higher average daily caloric intake (1,448 [236] vs 1,299 [285] kcal; *p* = 0.003) and a trend toward fewer days achieving the calorie goal of ≤1,200–1,500 kcal/day (215 [80] vs 245 [67] days; *p* = 0.05). Protein intake sufficiency (292.5 [26.3] vs 293.1 [25.3] days; *p* = 0.87) and dietary counselling attendance (11.9 [0.3] vs 11.8 [0.5] sessions; *p* = 0.45) were comparable between groups. These findings suggest that the prehabilitation group maintained higher overall physical activity levels at 12 months but did not demonstrate superior caloric restriction, which may partially explain the convergence in weight outcomes over time.

*Per-protocol analysis (Table SIV).* The per-protocol analysis, restricted to participants completing 12-month follow-up (prehabilitation: *n* = 57; standard care: *n* = 55), yielded results consistent with the ITT analysis. At 3 months, the prehabilitation group showed significantly greater TWL (23.6 [4.3] vs 20.7 ([1.4] kg; difference, 2.9 kg; 95% CI, 1.7 to 4.1; *p* < 0.001), higher %EWL (57.3% [11.5] vs 51.7% [12.9]; difference, 5.6%; *p* = 0.02), lower body fat (30.2% [3.8] vs 32.1% [4.1]; difference, −1.8%; *p* = 0.02), higher SF-36 physical score (65.6 [6.6] vs 62.8 [7.9]; difference, 2.9; *p* = 0.04), and higher SF-36 mental score (66.0 [7.9] vs 62.9 [7.4]; difference, 3.0; *p* = 0.04). As in the ITT analysis, all between-group differences attenuated and became non-significant by 6 and 12 months. The convergence of ITT and per-protocol results supports the robustness of the findings and suggests that loss to follow-up did not substantively bias the primary analysis.

*Subgroup analysis by surgery type (Table SV).* The subgroup analysis stratified by type of bariatric surgery revealed consistent patterns across both RYGB and SG subgroups. Among SG patients, the 3-month advantage of prehabilitation was more pronounced for TWL (24.7 [3.8] vs 20.7 [1.5] kg; difference, 4.0 kg; *p* < 0.001) compared with RYGB patients (22.6 [4.5] vs 20.7 [1.4] kg; difference, 1.9 kg; *p* = 0.03). By 12 months, no significant between-group differences persisted in either the RYGB or SG subgroup for TWL, %EWL, or BMI. Formal interaction testing confirmed that the type of bariatric surgery did not significantly modify the treatment effect at 12 months (interaction *p*-values: TWL, *p* = 0.46; %EWL, *p* = 0.50; BMI, *p* = 0.60). These findings suggest that the pattern of early prehabilitation benefit with subsequent convergence is consistent regardless of surgical procedure.

## DISCUSSION

In this propensity score matched retrospective cohort study, a 6-week multimodal prehabilitation programme did not produce a statistically significant improvement in total weight loss at 12 months compared with standard preintervention education in patients undergoing metabolic and bariatric surgery. The between-group difference in the primary outcome was 0.3 kg, which was neither statistically significant nor clinically meaningful. This finding was consistent across the unadjusted analysis, the adjusted linear mixed-effects model, and the per-protocol sensitivity analysis.

The study did, however, reveal a notable temporal pattern. Our results show that prehabilitation increased total weight loss by 2.85 kg compared with standard care at the 3-month follow-up (adjusted mean difference; 95% CI, 1.77 to 3.93; *p* < 0.001) (27). This early advantage extended to several secondary outcomes, including percentage excess weight loss, BMI, body fat percentage, diastolic blood pressure, grip strength, and the SF-36 mental component score. All of these differences were attenuated by 6 months and were no longer detectable at 12 months. This pattern suggests that the preoperative conditioning provided by prehabilitation accelerates early postoperative recovery and weight loss, but that the dominant effect of the surgical procedure itself equalizes the trajectories over time. The bariatric operation is a powerful metabolic intervention (3, 4), and its effects on appetite regulation, gut hormone signalling, and energy metabolism may eventually override the incremental physiological gains achieved through a 6-week preoperative programme (28).

These findings diverge from the prehabilitation literature in colorectal cancer surgery, where multimodal programmes have demonstrated sustained reductions in postoperative complications and shorter hospital stays (9, 10). The PREHAB trial reported a 53% reduction in severe complications following colorectal resection (11). The discordance likely reflects fundamental differences between the 2 surgical populations. Bariatric patients are generally younger and carry a lower burden of frailty than patients undergoing colorectal cancer resection (7). The physiological reserve available for recovery is therefore greater, and the ceiling for improvement from prehabilitation may be lower. Moreover, the primary outcome in bariatric surgery is weight loss over months and years, not short-term surgical morbidity (29). The bariatric outcome is driven by the anatomy created at surgery and by sustained postoperative behavioural change (6). A 6-week preoperative intervention may be insufficient to alter these long-term determinants.

The economic findings should be interpreted with caution. The prehabilitation group incurred an additional cost of ¥6,792 per patient, due almost entirely to the prehabilitation programme fee, while the surgery-related costs that dominate total expenditure were essentially identical between groups. Because prehabilitation produced no statistically significant difference in 12-month effectiveness, and because the single cost difference (the programme fee) carries no statistical uncertainty, a formal cost-effectiveness analysis is uninformative here; our data therefore provide no evidence that prehabilitation is cost-effective and do not support recommending it on cost-effectiveness grounds, although they also cannot exclude the possibility that it is cost-effective over a longer horizon. We did not compute a cost-per-quality-adjusted-life-year (QALY) ratio. We note that this was a deliberate choice rather than a data limitation: because the SF-6D preference-based utility index can be derived from 6 items of the SF-36, the necessary information was in principle available. We elected not to pursue a cost-utility analysis because it would not change the conclusion – the effectiveness differences were confined to the 3-month time point and did not persist to 12 months, so any utility gain would be similarly transient – and because a robust cost-utility analysis would require a longer time horizon than the present study affords. A formal cost-utility analysis using SF-6D-derived utilities, ideally with longer follow-up, is nonetheless a worthwhile direction for future research (13–15).

On a more positive note, the safety profile of the prehabilitation programme was favourable. No serious adverse events occurred in either group throughout the study period. The prehabilitation group also demonstrated significantly greater physical activity levels at 12 months, including higher average weekly exercise and more complete activity logs (30). These behavioural differences, while encouraging, did not translate into better weight outcomes. The dietary adherence data added nuance: the prehabilitation group had higher daily caloric intake at 12 months and fewer days meeting the calorie restriction target. This unexpected finding may reflect a complex interplay between increased exercise-related energy expenditure and compensatory eating behaviour, a phenomenon documented in the exercise physiology literature (31). It may also suggest that the prehabilitation programme was more effective at establishing exercise habits than at sustaining dietary discipline (32).

### Limitations

Several limitations warrant consideration in the interpretation of these findings. First, despite propensity score matching on 31 baseline covariates with all standardized mean differences below 0.1 (25), residual confounding by unmeasured factors cannot be excluded. Patients who enrolled in prehabilitation may have differed from the control pool in motivation, health literacy, or social support in ways not captured by the measured variables. Second, although the treatment and control groups were drawn from the same institution over the same overall enrolment window, the prehabilitation group comprised consecutive programme enrolees, so the 2 groups were not sampled from identical calendar periods and may differ with regard to unmeasured time-varying factors, such as secular changes in perioperative care or seasonal variation in physical activity and bodyweight, which propensity score matching cannot address (42). We consider it unlikely that such factors explain the observed pattern of an early (3-month) benefit that disappeared by 12 months, because any seasonal or secular influence would be expected to act on both arms and across the full follow-up rather than to produce a transient early divergence; nonetheless, this possibility cannot be formally excluded. Third, the retrospective observational design cannot establish causality with the same confidence as a randomized trial; propensity score matching adjusts for measured confounders but cannot account for unmeasured differences between groups (16). Fourth, the study was conducted at a single institution in China, and the results may not generalize to other settings, surgical teams, or patient populations. Fifth, the prehabilitation programme lasted 6 weeks; longer or more intensive programmes might produce different results (12). Sixth, the 12-month follow-up, while aligned with standard bariatric outcome reporting (29), may be insufficient to detect delayed or cumulative benefits. Seventh, the economic analysis captured costs at 3 months and did not incorporate preference-based utilities or quality-adjusted life-years; because the SF-36 is a health-profile instrument rather than a preference-based measure, deriving utilities would require a validated mapping algorithm (for example, to the SF-6D) and value set that were beyond the scope of the present data, and a formal cost–utility analysis is an important direction for future work. Finally, the sample size of 60 per group provides limited statistical power to detect small but potentially meaningful differences, particularly in secondary outcomes.

In conclusion, this study found that a 6-week multimodal prehabilitation programme accelerated early weight loss and improved several functional and patient-reported outcomes at 3 months following metabolic and bariatric surgery, but these early advantages did not persist at 12 months. Prehabilitation did not improve the primary outcome of total weight loss at 12 months, and because it added programme cost without a demonstrable difference in the primary outcome, a cost-effectiveness advantage could not be established. Future research should examine whether longer prehabilitation programmes, alternative intervention designs, or more targeted patient selection can produce durable benefits, and should incorporate longer follow-up and formal cost–utility analysis to determine whether the concept of prehabilitation, which has demonstrated clear value in other surgical contexts (9, 11), can be effectively adapted for metabolic and bariatric surgery.

## Supplementary Material


