# Supplementary material for: PREHABILITATION FOR PATIENTS UNDERGOING METABOLIC/BARIATRIC SURGERY: A RETROSPECTIVE COHORT STUDY USING PROPENSITY SCORE MATCHING
Source: J Rehabil Med. 2026 Aug 10;58:45736. doi: 10.2340/jrm.v58.45736 (PMC13463913; doi:10.2340/jrm.v58.45736)
Supplement: Supplementary file 1 [file JRM-58-45736-s1.pdf]

Supplementary Materials

Participants

Exclusion Criteria

Patients were excluded if they had undergone prior bariatric or major upper gastrointestinal surgery; had a planned revisional bariatric procedure; were classified as ASA IV or higher; had uncontrolled cardiac arrhythmia (including atrial fibrillation with rapid ventricular response) or unstable angina; had severe cardiopulmonary disease contraindicating supervised exercise (NYHA class III to IV heart failure or oxygen-dependent COPD); had severe anemia (hemoglobin less than 10 g/dL); had active systemic infection or acute inflammation (CRP greater than 20 mg/L at screening); met DSM-5 criteria for active substance use disorder; had uncontrolled psychiatric illness precluding safe participation and informed consent; had a musculoskeletal or neurological condition preventing participation in the exercise component; were pregnant or planning pregnancy within 12 months; had end-stage renal disease requiring dialysis; or had active malignancy under treatment. Participants aged 16 to 17 years required additional written parental or guardian consent.

Allocation Mechanism

The prehabilitation program was introduced as a new clinical care pathway beginning in November 2021. Consecutive patients meeting the eligibility criteria and scheduled for primary metabolic or bariatric surgery were offered enrollment in the prehabilitation program on the basis of the following pragmatic criteria: (1) surgical scheduling that allowed a minimum of 8 weeks between the initial consultation and the planned operative date, thereby permitting completion of the prehabilitation program; (2) geographic residence and logistical circumstances permitting regular attendance at supervised exercise and counseling sessions at the hospital facility; and (3) patient willingness to participate in the structured program. Patients who could not fulfill these conditions, owing to scheduling constraints, travel distance, employment commitments, or personal preference, received the institution's standard preintervention education and served as the concurrent control group.

The type of bariatric surgery (RYGB or SG) was determined independently of group assignment by the multidisciplinary bariatric team based on individual patient anatomy, comorbidity profile, and shared decision-making with the patient, following institutional guidelines. All surgical procedures were performed by the same surgical team using standardized operative techniques and perioperative protocols, regardless of whether the patient had received prehabilitation or standard care. Postoperative management, including dietary advancement, follow-up scheduling, and activity recommendations, was identical for both groups.

The Prehabilitation Program

Program Overview

The prehabilitation program was delivered over 6 consecutive weeks prior to bariatric surgery. It comprised three integrated components: supervised exercise training, individualized nutritional counseling, and group-based psychological support. The program was designed following the trimodal prehabilitation model pioneered by Carli et al.[1,2] at McGill University. It was adapted to address the unique physiological, metabolic, and psychosocial challenges of patients with severe obesity undergoing metabolic surgery.[3,4,5]

The program was supervised by a multidisciplinary team. This team included a certified exercise physiologist, a registered dietitian with bariatric experience, a clinical psychologist trained in cognitive-behavioral therapy, and a bariatric surgery coordinator. The exercise physiologist conducted all exercise sessions. The dietitian provided weekly individual nutritional consultations. The psychologist led weekly group-based psychological support sessions. The surgery coordinator-maintained adherence logs and served as the liaison between the prehabilitation team and the surgical service.

Component 1: Supervised Exercise Training

Frequency: 3 supervised sessions per week. Duration: 60 minutes per session. Total: 18 sessions over 6 weeks. Setting: hospital-based exercise facility. Supervision: certified exercise physiologist present at all sessions. Heart rate monitoring: continuous telemetry using a chest-strap heart rate monitor.

The aerobic component targeted the cardiorespiratory system. Modalities included treadmill walking and stationary cycling, selected on the basis of patient preference and joint tolerance. Intensity was prescribed using heart rate reserve calculated from resting heart rate and age-predicted maximal heart rate.[6,8] Perceived exertion was used as a complementary guide. The resistance component targeted six major muscle groups using weight machines. One-repetition maximum was estimated at baseline using the submaximal prediction method. Load progression followed the principle of progressive overload: when the patient completed all prescribed repetitions across two consecutive sessions, the load was increased by approximately 5 percent.[7,9]

Week	Aerobic Component	Intensity	RPE Target	Resistance Sets x Reps	Resistance Load	Warm-up / Cool-down
**1**	Treadmill or cycle, 20 min continuous	50% HRR	11 to 12	2 x 10	50% est. 1RM	5 min walk + stretching each
**2**	Treadmill or cycle, 25 min continuous	50% HRR	12	2 x 12	50 to 55% est. 1RM	5 min walk + stretching each
**3**	Treadmill or cycle, 25 min; add 5 min interval block	60% HRR	12 to 13	2 x 12 to 15	60% est. 1RM	5 min walk + stretching each
**4**	Treadmill or cycle, 30 min with 2 interval blocks	60% HRR	13	3 x 12	60 to 65% est. 1RM	5 min walk + stretching each
**5**	Treadmill or cycle, 30 min with 3 interval blocks	70% HRR	13 to 14	3 x 12 to 15	65 to 70% est. 1RM	5 min walk + stretching each
**6**	Treadmill or cycle, 30 min with 3 interval blocks	70% HRR	14	3 x 15	70% est. 1RM	5 min walk + stretching each

Supplementary Table. Week-by-week exercise prescription. HRR = heart rate reserve. RPE = rating of perceived exertion on the Borg 6 to 20 scale. 1RM = one-repetition maximum. Interval blocks consisted of 2-minute higher-intensity bouts at the upper end of the target range alternating with 2-minute recovery periods. The six resistance exercises were: chest press, seated row, leg press, leg curl, shoulder press, and abdominal crunch. All exercises were performed on weight machines to

ensure safety and standardization. Seated or recumbent alternatives were used when standing exercises provoked excessive joint discomfort.

Component 2: Individualized Nutritional Counseling

Frequency: 1 individual session per week with a registered dietitian. Duration: 30 to 45 minutes per session. Total: 6 sessions over 6 weeks.

Week	Caloric Target	Protein Target	Dietary Format	Counseling Focus
**1**	1200 to 1500 kcal/day	Min 60 g/day	Balanced solid diet	Baseline dietary assessment. Identify current patterns and barriers. Introduce caloric restriction rationale. Set initial meal plan.
**2**	1200 to 1500 kcal/day	Min 60 g/day	Balanced solid diet	Portion control training. Label reading workshop. Review of food diary from week 1. Address adherence challenges.
**3**	1200 to 1500 kcal/day	Min 60 g/day	Balanced solid diet with protein-enriched meals	Introduction to high-protein food sources. Meal preparation strategies. Hydration targets of at least 1.5 L per day, separating fluids from meals.
**4**	1200 to 1500 kcal/day	Min 60 g/day	Protein-enriched diet; introduce protein supplement if needed	Transition planning toward postoperative diet stages. Practice bariatric plate method. Review micronutrient supplementation protocol.
**5**	800 kcal/day (VLCD)	Min 80 g/day	Liquid-predominant VLCD: protein shakes, broth, sugar-free liquids	Initiation of liver-shrinkage diet. Education on VLCD rationale for hepatic volume reduction and improved surgical access. Managing hunger and energy during VLCD.
**6**	800 kcal/day (VLCD)	Min 80 g/day	Liquid-predominant VLCD continued through to surgery	Final preoperative dietary review. Detailed walkthrough of postoperative diet stages: clear liquids, full liquids, pureed, soft, regular. Reinforce lifelong dietary principles.

VLCD = very-low-calorie diet. The VLCD in weeks 5 and 6 was designed to reduce hepatic steatosis and intrahepatic fat, thereby decreasing liver volume and facilitating laparoscopic surgical exposure.[12,13] Commercially available protein shakes providing 25 to 30 g of whey protein per serving were used as the primary caloric source during the VLCD phase. Patients who could not meet the 80 g per day protein target through shakes alone received additional whey protein isolate supplementation. Micronutrient supplementation was initiated from week 1 in accordance with the AACE/TOS/ASMBS guidelines: a daily bariatric-formulated multivitamin, calcium citrate 1200 to 1500 mg per day in divided doses, vitamin D3 3000 IU per day, and elemental iron 45 to 60 mg per day for menstruating women.[3,4]

Component 3: Group-Based Psychological Support

Frequency: 1 group session per week. Group size: 8 to 12 patients per group. Duration: 60 to 90 minutes per session. Total: 6 sessions over 6 weeks. Facilitated by a clinical psychologist trained in cognitive-behavioral therapy and motivational interviewing.

Week	Session Theme	Key Activities and Techniques	Take-Home Task
**1**	Psychoeducation: Understanding Emotional Eating	Introduction to the CBT model of eating behavior. Group discussion on links between emotions, thoughts, and eating. Identification of personal triggers.	Begin daily food and mood diary. Record all eating episodes with associated emotional state.
**2**	Goal Setting and Motivation	Motivational interviewing exercises: exploring values, weighing pros and cons, envisioning life after surgery. Collaborative setting of realistic preoperative goals.	Write a personal motivation letter. Identify three specific behavioral goals for the program.
**3**	Cognitive Restructuring: Beliefs About Food and Body	Identifying automatic negative thoughts related to food, body image, and self-worth. Thought challenging exercises. Introduction to alternative balanced thoughts.	Complete thought record worksheets when noticing urges to overeat or negative body-related thoughts.
**4**	Self-Monitoring and Behavioral Activation	Review of food and mood diaries. Behavioral activation techniques: scheduling pleasurable activities unrelated to food. Stimulus control strategies for the home food environment.	Implement one stimulus control change at home. Schedule two non-food pleasurable activities this week.
**5**	Coping with Perioperative Anxiety	Psychoeducation on normal perioperative anxiety. Diaphragmatic breathing and progressive muscle relaxation training. Guided imagery for surgical preparation.	Practice relaxation technique daily for 10 minutes. Record anxiety levels before and after practice.
**6**	Relapse Prevention and Postoperative Preparation	Identifying high-risk situations for dietary non-adherence after surgery. Developing a personalized coping plan. Consolidating skills learned across the program. Open discussion of fears and expectations.	Complete a written relapse prevention plan with identified risks, warning signs, and specific coping responses.

The psychological component drew on established evidence for preoperative cognitive-behavioral therapy in bariatric patients.[14,15,16] Motivational interviewing principles were woven through all sessions. The psychologist used open-ended questions, affirmations, reflective listening, and summaries to support autonomous motivation and resolve ambivalence.[17] Patients scoring above 11 on either the HADS anxiety or depression subscale at baseline were offered one additional individual session during week 3 or 4 for targeted assessment and support. This individual session served as a safety net,

not a replacement for the group program. Patients with scores indicating severe distress were referred for formal psychiatric evaluation.

Adherence Monitoring

Program adherence was tracked through four mechanisms. First, attendance at all supervised sessions was recorded by the surgery coordinator. A minimum attendance of 80 percent of sessions across all three components was defined as adequate adherence. Second, the exercise physiologist recorded each patient's aerobic duration, heart rate, resistance load, and repetitions at every session. Third, patients submitted weekly food diaries and protein intake logs, which the dietitian reviewed during each counseling session. Fourth, patients completed a brief weekly check-in questionnaire documenting self-rated adherence to the caloric and protein targets, exercise completed outside of supervised sessions, and use of the psychological techniques practiced in group sessions. Body weight was measured weekly under standardized conditions to monitor trajectory.

Safety Considerations and Modifications for Severe Obesity

All exercise sessions were conducted under continuous heart rate monitoring. Blood pressure was measured before and after each session. The exercise physiologist was trained to recognize warning signs including chest pain, excessive dyspnea, dizziness, and abnormal heart rate responses. Patients were instructed to report any new symptoms between sessions. Joint-protective modifications were standard: recumbent cycling was used in preference to treadmill walking for patients with significant knee or hip discomfort, and all resistance exercises were performed on machines rather than free weights to ensure stability and reduce fall risk. The VLCD phase in weeks 5 and 6 was monitored by the dietitian with attention to hydration status, electrolyte symptoms, and blood glucose in patients with prediabetes or type 2 diabetes. Patients on glucose-lowering medications received individualized medication adjustment by their attending endocrinologist during the VLCD phase.[10,12]

Standard Preintervention Education Group

Patients in the control group received standard preintervention education in accordance with the institutional bariatric surgery protocol. This protocol reflected the multidisciplinary preoperative preparation recommended by the AACE/TOS/ASMBS clinical practice guidelines[3,4] and the Enhanced Recovery After Surgery Society bariatric recommendations.[5] The program consisted of two structured preoperative counseling sessions delivered within the 6 weeks preceding surgery. No supervised exercise, formal behavioral therapy, or extended lifestyle intervention was provided.

Session 1: Dietary and Nutritional Counseling

The first session was a one-on-one consultation with a registered dietitian, lasting approximately 45 to 60 minutes. It was scheduled 4 to 6 weeks before the planned operative date. The session served two purposes. The first was to provide dietary education. The dietitian reviewed the rationale for preoperative caloric restriction and explained the postoperative diet progression through its standard phases: clear liquids, full liquids, pureed foods, soft foods, and a regular texture-modified diet. Patients received written guidance on portion control, adequate hydration, and the importance of chewing thoroughly. The dietitian emphasized a protein intake target of at least 60 g per day and reviewed food sources rich in lean protein. The

second purpose was to initiate the preoperative liver-shrinkage diet. Patients were instructed to follow a low-calorie, high-protein liquid diet of approximately 800 kcal per day during the final 2 weeks before surgery, consistent with standard institutional practice for reducing hepatic volume and improving intraoperative visualization.

The dietitian also screened for existing micronutrient deficiencies and initiated preoperative supplementation where indicated: a daily bariatric-formulated multivitamin, calcium citrate, vitamin D, and iron for at-risk patients. Patients received a printed dietary booklet summarizing the preoperative and postoperative dietary stages, recommended food choices, and a sample 7-day meal plan for the liquid diet phase. No additional dietitian follow-up sessions were scheduled prior to surgery.

Session 2: Surgical Preparation and General Lifestyle Guidance

The second session was a group-based educational seminar, lasting approximately 90 minutes. It took place 2 to 4 weeks before surgery and was co-facilitated by the bariatric surgeon and a bariatric nurse coordinator. The session was attended by 8 to 15 patients per group.

The surgical preparation component covered what to expect on the day of surgery, the general steps of the planned procedure, anticipated length of hospital stay, wound care instructions, and signs and symptoms requiring emergency medical attention. The surgeon discussed the expected trajectory of weight loss and the realistic timeframe for results. Informed consent was reviewed. Patients were given the opportunity to ask questions. The nurse coordinator outlined the perioperative logistics: fasting instructions, medication adjustments, venous thromboembolism prophylaxis, and the postoperative follow-up schedule at 3, 6, and 12 months.

The lifestyle guidance component was brief. Patients were advised to gradually increase their daily walking and to reduce prolonged sedentary time in the weeks before surgery. A general recommendation of at least 150 minutes of moderate-intensity physical activity per week was communicated verbally, consistent with public health guidelines. However, no individualized exercise prescription was provided. No supervised training sessions were offered. No exercise log or monitoring system was used. This reflects the typical practice in bariatric programs, where physical activity counseling is delivered briefly and without formal structure.

The psychological component was limited to a single preoperative screening assessment. All patients underwent a standardized psychosocial evaluation by a clinical psychologist as part of the routine surgical workup. This evaluation assessed readiness for surgery, screened for uncontrolled psychiatric illness and active eating disorders, and documented the patient's understanding of the required postoperative behavioral changes. It was diagnostic in nature. It did not include therapeutic intervention, cognitive-behavioral techniques, or motivational interviewing. Patients identified as having significant psychological concerns were referred for further evaluation before surgical clearance was granted.

What the Standard Group Did Not Receive

To delineate the contrast with the prehabilitation group, the following elements were explicitly absent from the standard preintervention education protocol: no supervised aerobic or resistance exercise sessions; no progressive exercise prescription; no weekly dietitian follow-up beyond the single initial consultation; no group-based or individual cognitive-behavioral therapy; no motivational interviewing; no structured anxiety management training; no relaxation or coping skills instruction; no adherence monitoring system for exercise, diet, or psychological homework; and no formal prehabilitation

program of any kind. This description is consistent with what published bariatric surgery trials have defined as standard or usual presurgical care.

Postoperative Care

After surgery, patients in both the standard education group and the prehabilitation group received identical postoperative management. This included the same dietary advancement protocol progressing from clear liquids on postoperative day 1 through full liquids, pureed foods, soft foods, and a regular texture-modified diet over 6 to 8 weeks. All patients received the same postoperative supplementation regimen. Scheduled follow-up visits occurred at 3, 6, and 12 months and included clinical assessment, laboratory testing, anthropometric measurements, functional testing, and administration of patient-reported outcome instruments. All postoperative visits were conducted by the same clinical team regardless of group assignment.

Propensity Score Matching

Covariates for the propensity score model were selected a priori on clinical and theoretical grounds, not by data-driven or stepwise selection. Consistent with established guidance, we prioritized variables that are prognostic for the outcome (postoperative weight loss) or associated with both treatment assignment and outcome, because including outcome-related covariates reduces confounding bias and improves the precision of the estimated treatment effect, whereas variables related only to treatment (instruments) were avoided. The following 31 baseline covariates were included as independent variables in the propensity score logistic regression model: age, sex, body mass index, type of bariatric surgery (RYGB vs SG), education level, insurance type, current smoking status, current alcohol use, C-reactive protein level, albumin level, hemoglobin level, presence of hypertension, presence of cardiovascular disease, presence of obstructive sleep apnea, presence of gastroesophageal reflux disease, body weight, systolic blood pressure, diastolic blood pressure, body fat percentage, waist circumference, fasting glucose, total cholesterol, LDL cholesterol, HDL cholesterol, triglycerides, 6-minute walk test distance, grip strength, SF-36 physical component score, SF-36 mental component score, HADS anxiety score, and HADS depression score. The size of the standard-care candidate pool (254 patients) provided an adequate number of events per variable for stable estimation of the propensity score. We further note that, although 31 covariates is large relative to the 60 treated patients and exceeds the conventional 10-events-per-variable guideline for logistic regression, this guideline applies to models whose coefficients are interpreted for inference or prediction. Here the logistic model serves only to estimate a propensity score used for balancing, so a richer covariate set is acceptable; propensity score methods have been shown to be less biased and more precise than outcome logistic regression when events are few relative to the number of confounders. We assessed the adequacy of the estimated score by its achieved balance (all absolute standardized mean differences < 0.1; variance ratios within 0.8–1.25) and by confirming common support before matching, and we additionally adjusted for a parsimonious set of prognostic covariates within the matched sample to obtain a doubly robust estimate.

Statistical Analysis: Linear Mixed-Effects Model Diagnostics

The assumptions of the linear mixed-effects models used for the primary adjusted analyses were evaluated as follows. Normality of the conditional (within-patient) residuals was assessed visually using quantile-quantile plots and formally using the Shapiro-Wilk test. Homoscedasticity (constant residual variance) was assessed from

scatter plots of standardized residuals against fitted values and against time. The normality of the patient-level random intercepts was examined using quantile-quantile plots of the empirical best linear unbiased predictors (EBLUPs). Across the primary and secondary outcome models, residuals were approximately normally distributed and showed no systematic trend with fitted values, and the random intercepts were consistent with a normal distribution; minor departures at the distribution tails were observed for a small number of skewed outcomes. Linear mixed-effects models are known to be robust to mild violations of these distributional assumptions, and estimates of fixed-effect treatment coefficients and their standard errors are minimally affected by such departures. Model parameters were estimated by restricted maximum likelihood, and all available observations were used under the missing-at-random assumption without imputation.

Full Outcome Definitions

Total weight loss (TWL) was calculated as baseline body weight minus body weight at 12 months. Percent excess weight loss (%EWL) was calculated using an ideal BMI of 25 kg/m². Safety outcomes included any serious adverse event, specific surgical or device-related complications (gastrointestinal bleeding, device migration, gastrointestinal obstruction or perforation, hepatic abscess, pancreatitis), number of hospitalizations, number of emergency room visits related to the intervention, and additional procedures such as endoscopy for complications. Safety was assessed at 3, 6, and 12 months.

For the cost-effectiveness analysis, costs were classified into three categories: (1) direct medical costs, comprising the intervention (surgical procedure plus device procurement), prehabilitation program costs, follow-up visit costs, and complication management costs; (2) direct non-medical costs, including transportation to the hospital and dietary program costs borne out of pocket; and (3) indirect costs, comprising lost wages for participants and lost wages for family members. All costs were assessed at 3 months postsurgery and expressed in Chinese Yuan.

Exercise adherence measures included average weekly exercise minutes, number of weeks meeting the 150 minutes per week recommendation, and physical activity log completion rate. Dietary adherence measures included average daily caloric intake, number of days achieving calorie goals of 1200 to 1500 kcal per day, number of days with sufficient protein intake of 60 to 80 g per day, and attendance at dietary counseling sessions. Both adherence domains were assessed at 12 months.

REFERENCES

1. Carli F, Zavorsky GS. Optimizing functional exercise capacity in the elderly surgical population. Curr Opin Clin Nutr Metab Care 2005; 8: 23-32.
2. Li C, Carli F, Lee L, Charlebois P, Stein B, Liberman AS, et al. Impact of a trimodal prehabilitation program on functional recovery after colorectal cancer surgery: a pilot study. Surg Endosc 2013; 27: 1072-1082.
3. Mechanick JI, Youdim A, Jones DB, Garvey WT, Hurley DL, McMahon MM, et al. Clinical practice guidelines for the perioperative nutritional, metabolic, and nonsurgical support of the bariatric surgery patient--2013 update. Obesity (Silver Spring) 2013; 21 Suppl 1: S1-S27.
4. Mechanick JI, Apovian C, Brethauer S, Garvey WT, Joffe AM, Kim J, et al. Clinical practice guidelines for the perioperative nutrition, metabolic, and nonsurgical support of patients undergoing bariatric procedures - 2019 update. Surg Obes Relat Dis 2020; 16: 175-247.
5. Thorell A, MacCormick AD, Awad S, Reynolds N, Roulin D, Demartines N, et al. Guidelines for perioperative care in bariatric surgery: Enhanced Recovery After Surgery (ERAS) Society recommendations. World J Surg 2016; 40: 2065-2083.
6. Donnelly JE, Blair SN, Jakicic JM, Manore MM, Rankin JW, Smith BK. American College of Sports Medicine Position Stand. Appropriate physical activity intervention strategies for weight loss and prevention of weight regain for adults. Med Sci Sports Exerc 2009; 41: 459-471.
7. Baillot A, Mampuya WM, Dionne IJ, Comeau E, Meziat-Burdin A, Langlois MF. Impacts of supervised exercise training in addition to interdisciplinary lifestyle management in subjects awaiting bariatric surgery: a randomized controlled study. Obes Surg 2016; 26: 2602-2610.
8. Baillot A, Audet M, Baillargeon JP, Dionne IJ, Valiquette L, Rosa-Fortin MM, et al. Impact of physical activity and fitness in class II and III obese individuals: a systematic review. Obes Rev 2014; 15: 721-739.
9. Baillot A, Mampuya WM, Comeau E, Meziat-Burdin A, Langlois MF. Feasibility and impacts of supervised exercise training in subjects with obesity awaiting bariatric surgery: a pilot study. Obes Surg 2013; 23: 882-891.
10. Bond DS, Vithiananthan S, Thomas JG, Trautvetter J, Unick JL, Jakicic JM, et al. Bari-Active: a randomized controlled trial of a preoperative intervention to increase physical activity in bariatric surgery patients. Surg Obes Relat Dis 2015; 11: 169-177.
11. Gillis C, Buhler K, Bresee L, Carli F, Gramlich L, Culos-Reed N, et al. Effects of nutritional prehabilitation, with and without exercise, on outcomes of patients who undergo colorectal surgery: a systematic review and meta-analysis. Gastroenterology 2018; 155: 391-410.
12. Van Nieuwenhove Y, Dambrauskas Z, Campillo-Soto A, van Dielen F, Wiezer R, Janssen I, et al. Preoperative very low-calorie diet and operative outcome after laparoscopic gastric bypass: a randomized multicenter study. Arch Surg 2011; 146: 1300-1305.
13. Holderbaum M, Casagrande DS, Sussenbach S, Buss C. Effects of very low calorie diets on liver size and weight loss in the preoperative period of bariatric surgery: a systematic review. Surg Obes Relat Dis 2018; 14: 237-244.

14. Gade H, Friborg O, Rosenvinge JH, Småstuen MC, Hjelmesæth J. The impact of a preoperative cognitive behavioural therapy (CBT) on dysfunctional eating behaviours, affective symptoms and body weight 1 year after bariatric surgery: a randomised controlled trial. Obes Surg 2015; 25: 2112-2119.
15. Cassin SE, Sockalingam S, Du C, Wnuk S, Hawa R, Parikh SV. A pilot randomized controlled trial of telephone-based cognitive behavioural therapy for preoperative bariatric surgery patients. Behav Res Ther 2016; 80: 17-22.
16. David LA, Sijercic I, Cassin SE. Preoperative and post-operative psychosocial interventions for bariatric surgery patients: a systematic review. Obes Rev 2020; 21: e12926.
17. Kalarchian MA, Marcus MD, Courcoulas AP, Cheng Y, Levine MD. Preoperative lifestyle intervention in bariatric surgery: a randomized clinical trial. Surg Obes Relat Dis 2016; 12: 180-187.

Supplementary Table I. Complications at Months 3, 6, and 12

Time Point	Complication	Prehabilitation	Standard Care	P Value
**3 mo**	Any SAE, No. (%)	0 (0.0)	0 (0.0)	1.00
	Specific complications, No. (%)	0 (0.0)	0 (0.0)	—
	Hospitalizations, mean (SD)	1.00 (0.00)	1.00 (0.00)	1.00
	ER visits, mean (SD)	0.00 (0.00)	0.00 (0.00)	1.00
	Additional procedures, No. (%)	0 (0.0)	0 (0.0)	—
**6 mo**	Any SAE, No. (%)	0 (0.0)	0 (0.0)	1.00
	Specific complications, No. (%)	0 (0.0)	0 (0.0)	—
	Hospitalizations, mean (SD)	1.00 (0.00)	1.00 (0.00)	1.00
	ER visits, mean (SD)	0.00 (0.00)	0.00 (0.00)	1.00
	Additional procedures, No. (%)	0 (0.0)	0 (0.0)	—
**12 mo**	Any SAE, No. (%)	0 (0.0)	0 (0.0)	1.00
	Specific complications, No. (%)	0 (0.0)	0 (0.0)	—
	Hospitalizations, mean (SD)	1.00 (0.00)	1.00 (0.00)	1.00
	ER visits, mean (SD)	0.00 (0.00)	0.00 (0.00)	1.00
	Additional procedures, No. (%)	0 (0.0)	0 (0.0)	—

Abbreviations: SAE, serious adverse event; ER, emergency room. P values calculated using Fisher exact test for categorical and Mann-Whitney U test for continuous variables. No serious adverse events or specific complications were observed in either group during the study period.

Supplementary Table II. Adherence to Exercise at 12 Months

Measure	Prehabilitation (n=57)	Standard Care (n=55)	P Value
Average weekly exercise, min	136.5 (15.9)	99.6 (9.2)	<0.001
Weeks meeting ≥150 min/wk, No.	19.2 (11.2)	15.9 (4.8)	0.28
Physical activity logs completed, No.	126.8 (15.8)	105.3 (28.7)	<0.001

Values are mean (SD). P values calculated using Mann-Whitney U test. Per-protocol population with complete 12-month data.

Supplementary Table III Dietary Adherence at 12 Months

Measure	Prehabilitation (n=57)	Standard Care (n=55)	P Value
Average daily caloric intake, kcal	1448.0 (236.0)	1299.2 (284.9)	0.003
Days achieving calorie goal, No.	215.0 (79.8)	245.0 (66.7)	0.05
Days with sufficient protein, No.	292.5 (26.3)	293.1 (25.3)	0.87
Dietary counseling sessions, No.	11.9 (0.3)	11.8 (0.5)	0.45

Values are mean (SD). P values calculated using Mann-Whitney U test. Per-protocol population with complete 12-month data. Calorie goal defined as ≤1200–1500 kcal/day; protein sufficiency defined as ≥60–80 g/day.

Supplementary Table IV. Sensitivity Analysis: Per-Protocol Population

Outcome	Mo	Prehabilitation, Mean (SD)	Standard Care, Mean (SD)	Difference (95% CI)	P Value
Total weight loss, kg*	3	23.6 (4.3)	20.7 (1.4)	2.9 (1.7 to 4.1)	<0.001
	6	24.2 (5.0)	23.8 (1.4)	0.3 (−1.1 to 1.7)	0.64
	12	23.7 (5.1)	23.4 (2.7)	0.3 (−1.3 to 1.8)	0.75
%EWL	3	57.3 (11.5)	51.7 (12.9)	5.6 (0.9 to 10.2)	0.02
	6	58.5 (12.9)	59.2 (14.4)	−0.7 (−5.9 to 4.5)	0.79
	12	57.1 (12.9)	58.1 (12.5)	−1.0 (−5.7 to 3.7)	0.69
BMI, kg/m²	3	30.2 (2.4)	31.2 (3.2)	−1.0 (−2.1 to 0.0)	0.06
	6	30.0 (2.4)	30.2 (3.1)	−0.2 (−1.2 to 0.9)	0.74
	12	30.2 (2.3)	30.3 (3.0)	−0.1 (−1.1 to 0.9)	0.87
Body fat, %	3	30.2 (3.8)	32.1 (4.1)	−1.8 (−3.3 to −0.3)	0.02
	6	29.9 (2.7)	30.2 (3.7)	−0.3 (−1.5 to 0.9)	0.64
	12	30.3 (3.0)	30.3 (3.7)	−0.0 (−1.3 to 1.2)	0.94
SF-36 Physical	3	65.6 (6.6)	62.8 (7.9)	2.9 (0.1 to 5.6)	0.04
	6	65.5 (8.0)	65.4 (7.7)	0.1 (−2.9 to 3.0)	0.97
	12	70.4 (7.9)	70.5 (7.7)	−0.1 (−3.0 to 2.8)	0.92
SF-36 Mental	3	66.0 (7.9)	62.9 (7.4)	3.0 (0.1 to 5.9)	0.04
	6	65.7 (8.3)	65.1 (8.0)	0.6 (−2.4 to 3.7)	0.70
	12	70.8 (8.4)	70.1 (8.0)	0.7 (−2.3 to 3.8)	0.64
HADS Anxiety	3	12.1 (3.0)	13.0 (2.9)	−0.9 (−2.0 to 0.2)	0.12
	6	12.4 (2.7)	12.5 (2.7)	−0.1 (−1.1 to 1.0)	0.91
	12	10.3 (2.8)	10.5 (2.7)	−0.2 (−1.2 to 0.8)	0.71
HADS Depression	3	11.8 (2.9)	12.2 (2.8)	−0.4 (−1.5 to 0.7)	0.44
	6	12.2 (2.1)	12.1 (2.1)	0.1 (−0.7 to 0.9)	0.78
	12	10.3 (2.2)	10.1 (2.2)	0.2 (−0.6 to 1.0)	0.68

Per-protocol population: Prehabilitation n=57; Standard Care n=55 (patients with complete 12-month follow-up data)

*Primary outcome. Per-protocol analysis restricted to participants completing 12-month follow-up (57 prehabilitation, 55 standard care). Results are consistent with the ITT analysis, confirming robustness of findings.

Supplementary Table V. Subgroup Analysis by Type of Bariatric Surgery

Surgery	Outcome	Mo	Prehabilitation	Standard Care	Difference (95% CI)	P
**RYGB**	TWL, kg	3	22.6 (4.5)	20.7 (1.4)	1.9 (0.2 to 3.6)	0.03
		6	23.1 (4.9)	23.8 (1.4)	−0.7 (−2.6 to 1.1)	0.44
		12	22.7 (5.2)	23.0 (2.8)	−0.3 (−2.5 to 1.9)	0.78
	%EWL	3	56.7 (13.7)	50.7 (9.2)	6.0 (−0.0 to 12.0)	0.06
		6	58.1 (15.2)	58.5 (10.2)	−0.4 (−7.1 to 6.3)	0.91
		12	57.6 (15.5)	56.8 (11.3)	0.7 (−6.4 to 7.9)	0.84
	BMI, kg/m²	3	30.3 (2.6)	31.4 (3.3)	−1.2 (−2.7 to 0.4)	0.15
		6	30.1 (2.6)	30.2 (3.1)	−0.1 (−1.6 to 1.4)	0.88
		12	30.1 (2.6)	30.5 (3.3)	−0.4 (−2.0 to 1.2)	0.65
**SG**	TWL, kg	3	24.7 (3.8)	20.7 (1.5)	4.0 (2.5 to 5.5)	<0.001
		6	25.0 (5.0)	23.8 (1.4)	1.2 (−0.7 to 3.2)	0.22
		12	24.6 (4.9)	23.8 (2.6)	0.8 (−1.2 to 2.9)	0.42
	%EWL	3	57.5 (9.0)	52.1 (15.8)	5.4 (−1.3 to 12.1)	0.12
		6	57.9 (10.3)	59.3 (17.5)	−1.4 (−9.0 to 6.2)	0.72
		12	56.6 (10.1)	59.2 (13.7)	−2.5 (−8.7 to 3.6)	0.42
	BMI, kg/m²	3	30.2 (2.1)	31.2 (3.1)	−1.0 (−2.4 to 0.4)	0.16
		6	30.1 (2.2)	30.3 (3.0)	−0.2 (−1.6 to 1.2)	0.83
		12	30.3 (2.2)	30.1 (2.8)	0.2 (−1.1 to 1.5)	0.79

Abbreviations: RYGB, Roux-en-Y gastric bypass; SG, sleeve gastrectomy; TWL, total weight loss; %EWL, percent excess weight loss; BMI, body mass index. Values are mean (SD). P values from independent t tests. Interaction p-values for surgery type × group at 12 months: TWL p=0.46, %EWL p=0.50, BMI p=0.60, indicating no significant effect modification by surgery type.
